# ESCRT recruitment to SARS-CoV-2 spike induces virus-like particles that improve mRNA vaccines

**DOI:** 10.1016/j.cell.2023.04.024

**Published:** 2023-05-25

**Authors:** Magnus A.G. Hoffmann, Zhi Yang, Kathryn E. Huey-Tubman, Alexander A. Cohen, Priyanthi N.P. Gnanapragasam, Leesa M. Nakatomi, Kaya N. Storm, Woohyun J. Moon, Paulo J.C. Lin, Anthony P. West, Pamela J. Bjorkman

**Affiliations:** 1 Division of Biology and Biological Engineering, California Institute of Technology, Pasadena, CA 91125, USA; 2 Acuitas Therapeutics, Vancouver, BC V6T 1Z3, Canada

**Keywords:** nanoparticles, mRNA vaccines, SARS-CoV-2, ESCRT

## Abstract

Prime-boost regimens for COVID-19 vaccines elicit poor antibody responses against Omicron-based variants and employ frequent boosters to maintain antibody levels. We present a natural infection-mimicking technology that combines features of mRNA- and protein nanoparticle-based vaccines through encoding self-assembling enveloped virus-like particles (eVLPs). eVLP assembly is achieved by inserting an ESCRT- and ALIX-binding region (EABR) into the SARS-CoV-2 spike cytoplasmic tail, which recruits ESCRT proteins to induce eVLP budding from cells. Purified spike-EABR eVLPs presented densely arrayed spikes and elicited potent antibody responses in mice. Two immunizations with mRNA-LNP encoding spike-EABR elicited potent CD8^+^ T cell responses and superior neutralizing antibody responses against original and variant SARS-CoV-2 compared with conventional spike-encoding mRNA-LNP and purified spike-EABR eVLPs, improving neutralizing titers >10-fold against Omicron-based variants for 3 months post-boost. Thus, EABR technology enhances potency and breadth of vaccine-induced responses through antigen presentation on cell surfaces and eVLPs, enabling longer-lasting protection against SARS-CoV-2 and other viruses.

## Highlights

• EABR technology engineers membrane proteins to induce assembly of enveloped VLPs
• SARS-CoV-2 spike-EABR eVLPs were genetically encoded and delivered as mRNA vaccine
• Spike-EABR mRNA elicits higher antibody titers than conventional spike mRNA in mice
• Spike-EABR mRNA elicits potent antibody responses against Omicron variants

## Graphical abstract

**Figure:**
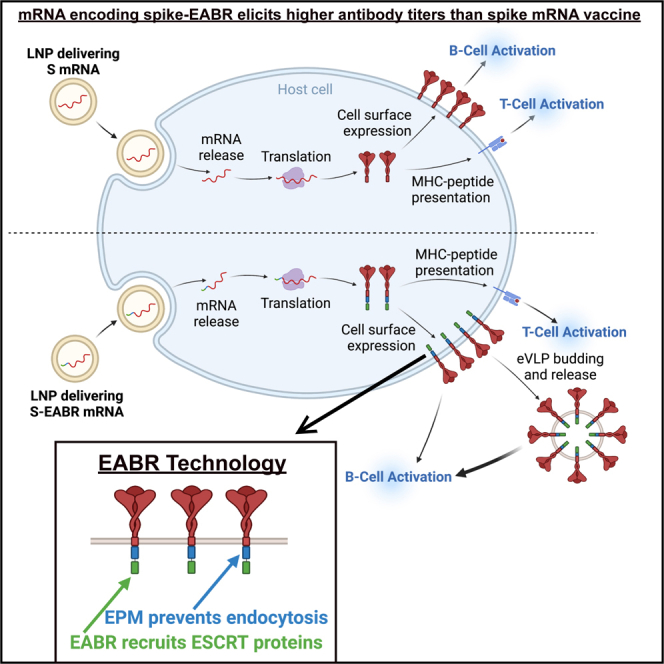

## Teaser

The SARS-CoV-2 spike protein was engineered to recruit ESCRT proteins to its cytoplasmic tail by adding an EABR motif, which induced the assembly of enveloped virus-like particles. A COVID-19 mRNA vaccine encoding spike-EABR elicited superior antibody responses compared with a conventional mRNA vaccine in mice.

## Introduction

mRNA vaccines emerged during the COVID-19 pandemic as an ideal platform for the rapid development of effective vaccines.^1^ Currently approved SARS-CoV-2 mRNA vaccines encode the viral spike (S) trimer,^2^ the primary target of neutralizing antibodies during natural infections.^3^ Clinical studies have demonstrated that mRNA vaccines are highly effective, preventing >90% of symptomatic and severe SARS-CoV-2 infections^4^^,^^5^ through both B and T cell responses.^6^ mRNA vaccines in part mimic an infected cell since expression of S within cells that take up S-encoding mRNAs formulated in lipid nanoparticles (LNP)^7^ results in cell surface expression of S protein to stimulate B cell activation. Translation of S protein inside the cell also provides viral peptides for presentation on MHC class I molecules to cytotoxic T cells, which does not commonly occur in protein nanoparticle-based vaccines^8^ that resemble the virus by presenting dense arrays of S protein; e.g., the Novavax NVX-CoV2373 vaccine.^9^^,^^10^ However, comparisons to COVID-19 mRNA vaccines showed that NVX-CoV2373 elicits comparable neutralizing antibody titers,^11^^,^^12^ a correlate of vaccine-induced protection,^13^ suggesting that potent B cell activation can be achieved through presentation of viral surface antigens on cell surfaces or virus-resembling nanoparticles. Achieving higher antibody neutralization titers is desirable as antibody levels contract substantially over a period of several months,^11^ and SARS-CoV-2 variants of concern (VOCs) that are less sensitive to antibodies elicited by vaccines or natural infection have been emerging.^14^^,^^15^^,^^16^ An optimal vaccine might therefore combine attributes of both mRNA- and protein nanoparticle-based vaccines by delivering a genetically encoded S protein that gets presented on cell surfaces and induces self-assembly and release of S-presenting nanoparticles.

Here, we describe a new technology that engineers membrane proteins to induce self-assembly of enveloped virus-like particles (eVLPs) that bud from the cell surface. This is accomplished for the SARS-CoV-2 S protein by inserting a short amino acid sequence (termed an endosomal sorting complex required for transport [ESCRT]- and ALG-2-interacting protein X [ALIX]-binding region or EABR)^17^ at the C terminus of its cytoplasmic tail to recruit host proteins from the ESCRT pathway. Many enveloped viruses recruit ESCRT-associated proteins such as TSG101 and/or ALIX through capsid or other interior viral structural proteins during the budding process.^18^^,^^19^ Thus, fusing the EABR to the cytoplasmic tail of a viral glycoprotein or other membrane protein directly recruits TSG101 and ALIX, bypassing the need for co-expression of other viral proteins for eVLP self-assembly. Cryoelectron tomography (cryo-ET) showed dense coating of spikes on purified S-EABR eVLPs, and direct injections of the eVLPs elicited potent neutralizing antibody responses in mice. Finally, we demonstrate that an mRNA vaccine encoding the S-EABR construct elicited at least 5-fold higher neutralizing antibody responses against SARS-CoV-2 and VOCs in mice than a conventional S-encoding mRNA vaccine or purified S-EABR eVLPs. These results demonstrate that mRNA-mediated delivery of S-EABR eVLPs elicits superior antibody responses, suggesting that dual presentation of viral surface antigens on cell surfaces and on extracellular eVLPs has the potential to enhance the effectiveness of COVID-19 mRNA vaccines.

## Results

### ESCRT recruitment to the spike cytoplasmic tail induces eVLP assembly

**Figure 1:**
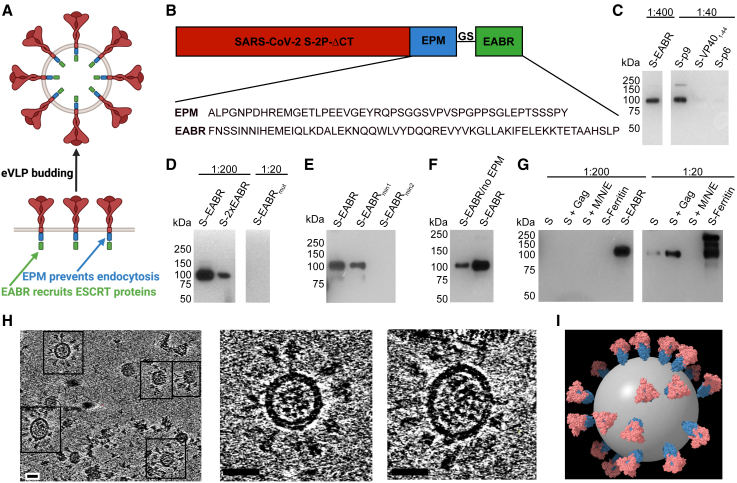
EABR insertion into the cytoplasmic tail of membrane proteins results in eVLP budding and release (A) Schematic of membrane-bound SARS-CoV-2 S proteins on the cell surface containing cytoplasmic tail EPM and EABR insertions that induce budding of an eVLP comprising a lipid bilayer with embedded S proteins. (B) Sequence information for S-EABR construct. Top: the SARS-CoV-2 S protein (including a furin cleavage site, 2P stabilizing substitutions, the D614G substitution, and ΔCT, a cytoplasmic tail deletion) is fused to an EPM sequence, a (Gly)_3_Ser (GS) spacer, and an EABR sequence. EPM, endocytosis prevention motif; GS, (Gly)_3_Ser linker; EABR, ESCRT- and ALIX-binding region. Bottom: EPM and EABR sequence information. (C–G) Western blot analysis detecting SARS-CoV-2 S1 protein on eVLPs purified by ultracentrifugation on a 20% sucrose cushion from transfected Expi293F cell culture supernatants. (C) Cells were transfected with S-EABR, S-p9, S-VP40_1–44_, or S-p6 constructs. The purified S-EABR eVLP sample was diluted 1:400 (left), while S-p9, S-VP40_1–44_, and S-p6 samples were diluted 1:40 (right). Comparison of band intensities between lanes suggests that the S-EABR eVLP sample contained ∼10-fold higher levels of S1 protein than the S-p9 sample and >10-fold higher levels than the S-VP40_1–44_ and S-p6 samples. (D) Cells were transfected with S-EABR, S-2xEABR (left), or S-EABR_mut_ constructs (right). Purified S-EABR and S-2xEABR eVLP samples were diluted 1:200, while the S-EABR_mut_ sample was diluted 1:20. (E) Cells were transfected with S-EABR, S-EABR_min1_, or S-EABR_min2_ constructs. Purified eVLP samples were diluted 1:200. (F) Cells were transfected with S-EABR/no EPM or S-EABR constructs. Purified eVLP samples were diluted 1:200. (G) Cells were transfected to express S alone, S plus the HIV-1 Gag protein, S plus the SARS-CoV-2 M, N, and E proteins, an S-ferritin fusion protein, or S-EABR. Purified eVLP samples were diluted 1:200 (left) or 1:20 (right). Comparison of band intensities between lanes suggests that the S-EABR eVLP sample contained >10-fold higher levels of S1 protein than S alone, S plus Gag, and S plus M, N, and E. (H) Computationally derived tomographic slices (8.1 nm) of S-EABR eVLPs derived from cryo-ET imaging of S-EABR eVLPs purified from transfected cell culture supernatants by ultracentrifugation on a 20% sucrose cushion and SEC. Left: representative eVLPs are highlighted in boxes. Middle and right: close ups of individual eVLPs. Scale bars, 30 nm. (I) Model of a representative S-EABR eVLP derived from a cryo-ET reconstruction (Video S1). Coordinates of an S trimer (PDB: 6VXX)^27^ were fit into protruding density on the best resolved half of an eVLP and the remainder of the eVLP was modeled assuming a similar distribution of trimers. The position of the lipid bilayer is shown as a 55-nm gray sphere. See also Figure S1 and Video S1.

**Figure S1:**
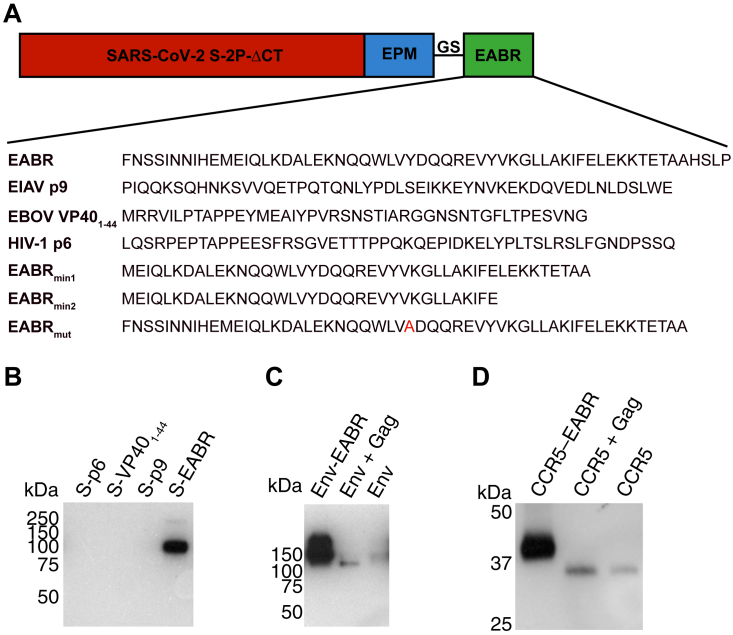
Comparison of EABR-related sequence insertions in the cytoplasmic tail of SARS-CoV-2 S, related to Figure 1 (A) Top: schematic of different S-EABR constructs that were compared for their ability to induce eVLP assembly. EPM, endocytosis prevention motif; GS, (Gly)_3_Ser linker; EABR, ESCRT- and ALIX-binding region. Bottom: amino acid sequences of EABR portion of different constructs. (B) Western blot analysis of SARS-CoV-2 S1 protein levels on eVLPs purified by ultracentrifugation on a 20% sucrose cushion from transfected Expi293F cell culture supernatants. Cells were transfected with S-p6, S-VP40_1–44_, S-p9, or S-EABR constructs. Purified eVLP samples were diluted 1:400. (C) Western blot analysis comparing HIV-1 Env_YU2_ levels in eVLP samples purified from transfected Expi293F cell culture supernatants. Cells were transfected with plasmids encoding Env-EABR, Env plus HIV-1 Gag, or Env alone. Purified eVLP samples were diluted 1:200. (D) Western blot analysis comparing CCR5 levels in eVLP samples purified from transfected Expi293F cell culture supernatants. Cells were transfected with plasmids encoding CCR5-EABR, CCR5 plus HIV-1 Gag, or CCR5 alone. Purified eVLP samples were diluted 1:200. The migration difference between CCR5-EABR and CCR5 is due to addition of the EABR sequence (∼7 kDa) that increases its molecular mass.

To evaluate the hypothesis that direct recruitment of ESCRT proteins to the cytoplasmic tail of a SARS-CoV-2 S protein could result in self-assembly and budding of eVLPs, we fused EABRs derived from different sources to the truncated cytoplasmic tail of the S protein, separated from its C terminus by a short Gly-Ser linker (Figures 1A and 1B). The S protein contained the D614G substitution,^20^ a furin cleavage site, two proline substitutions (2P) in the S2 subunit to stabilize the prefusion conformation,^21^ and the C-terminal 21 residues were truncated to optimize cell surface expression by removing an endoplasmic reticulum (ER)-retention signal (ΔCT)^22^ (Figure 1B). We evaluated the EABR fragment from the human CEP55 protein that binds TSG101 and ALIX during cytokinesis^17^ (Figure 1B). For comparisons, viral late domains that recruit early ESCRT proteins during the viral budding process were obtained from the Equine infectious anemia virus (EIAV) p9 protein,^23^ residues 1–44 of the Ebola virus (EBOV) VP40 protein,^24^ and the HIV-1 p6 protein^25^ (Figure S1A). We hypothesized that eVLP production could be enhanced by preventing endocytosis of EABR-fusion proteins to extend the duration that proteins remain at the plasma membrane to interact with ESCRT proteins. We therefore added an endocytosis prevention motif (EPM), a 47-residue insertion derived from the murine Fc gamma receptor FcgRII-B1 cytoplasmic tail (Figures 1A and 1B) that tethers FcgRII-B1 to the cytoskeleton to prevent coated pit localization and endocytosis.^26^

The abilities of the S-EABR, S-p9, S-VP40_1–44_, and S-p6 constructs to generate eVLPs were evaluated by transfecting Expi293F cells and measuring eVLP production in supernatants from which eVLPs were purified by ultracentrifugation on a 20% sucrose cushion. Western blot analysis showed that the highest S protein levels were detected for the S-EABR construct, suggesting that the CEP55 EABR induced efficient self-assembly of S-containing eVLPs (Figures 1C and S1B). At a sample dilution of 1:400, the S-EABR construct produced a similarly intense band compared with the S-p9 construct at a 1:40 dilution, suggesting that S protein levels were ∼10-fold higher. The CEP55 EABR binds both ALIX and TSG101,^17^ whereas EIAV p9 only binds ALIX,^23^ suggesting that optimal recruitment of both ESCRT proteins is required for efficient eVLP assembly. The S-p6 and S-VP40_1–44_ samples contained little or no S protein, suggesting that eVLP assembly was inefficient, possibly resulting from lower affinities for ESCRT proteins (Figures 1C and S1B).

We further characterized the S-EABR construct by experimenting with different EABR sequences (Figure S1A), finding that addition of a second EABR domain (S-2xEABR) reduced eVLP production (Figure 1D). To investigate whether S-EABR eVLP assembly is dependent on ESCRT recruitment, we generated S-EABR_mut_ by substituting an EABR residue (Tyr187 in CEP55) that is essential for interacting with ALIX^17^ (Figure S1A). While the purified S-EABR eVLP sample produced an intense band at a 1:200 dilution, no band was detected for S-EABR_mut_ at a 1:20 dilution, suggesting that eVLP production was abrogated for S-EABR_mut_ and highlighting the importance of ALIX recruitment for eVLP assembly (Figure 1D). To identify the minimal EABR sequence required for eVLP assembly, we designed S constructs fused to the complete EABR domain (CEP55_170–213_), EABR_min1_ (CEP55_180–213_), and EABR_min2_ (CEP55_180–204_) (Figure S1A). While S-EABR eVLP yields were diminished for EABR_min2_, production efficiency was retained for EABR_min1_ (Figure 1E). To assess the effects of the EPM within the cytoplasmic tail of the S-EABR construct, we evaluated eVLP production for an S-EABR construct that did not include the EPM. Western blot analysis demonstrated that increased amounts of S protein were detected after eVLP purification from cells transfected with S-EABR compared with S-EABR/no EPM, suggesting that the EPM enhances eVLP production (Figure 1F).

We also compared the S-EABR construct with other eVLP approaches^28^ that require co-expression of S protein with structural viral proteins, such as HIV-1 Gag^29^ or the SARS-CoV-2 M, N, and E proteins.^30^ Western blot analysis showed that purified S-EABR eVLP fractions contained at least 10-fold more S protein than eVLPs produced by co-expression of S and Gag or S, M, N, and E (Figure 1G), suggesting that S-EABR eVLPs assemble and/or incorporate S proteins more efficiently than the other eVLP approaches. Purified S-EABR eVLPs also contained higher levels of S protein compared with S-ferritin nanoparticles purified from transfected cell supernatants, which have been shown to elicit potent immune responses in animal models^31^^,^^32^ (Figure 1G).

3D reconstructions derived from cryo-ET showed purified S-EABR eVLPs with diameters ranging from 40 to 60 nm that are surrounded by a lipid bilayer and the majority of which were densely coated with spikes (Figures 1H and 1I; Video S1). To estimate the number of S trimers, we counted trimer densities in ∼4 nm computational tomographic slices of individual eVLPs, finding ∼10–40 spikes per particle that were heterogeneously distributed on the surface of eVLPs. The upper limit of the number of spikes on eVLPs roughly corresponds to spike numbers on larger SARS-CoV-2 virions (>100 nm in diameter)^33^; thus, the spike densities on the majority of eVLPs exceed those on authentic viruses. Spikes on eVLPs were separated by distances of ∼20–26 nm (measured between the centers of trimer apexes) for densely coated particles (Figures 1H and 1I). To assess the general applicability of the EABR approach, we also generated EABR eVLPs for HIV-1 Env, which produced eVLPs with higher Env content than co-expression of Env and HIV-1 Gag (Figure S1C), and for the multi-pass transmembrane protein CCR5 (Figure S1D). Taken together, these results are consistent with efficient incorporation of S proteins into S-EABR eVLPs that are released from transfected cells and suggest that the EABR technology can be applied to a wide range of membrane proteins.

Video S1. Tomographic reconstruction of purified S-EABR eVLPs, related to Figure 1 Representative eVLPs are highlighted in boxes.

### S-EABR eVLPs induce potent antibody responses in immunized mice

**Figure 2:**
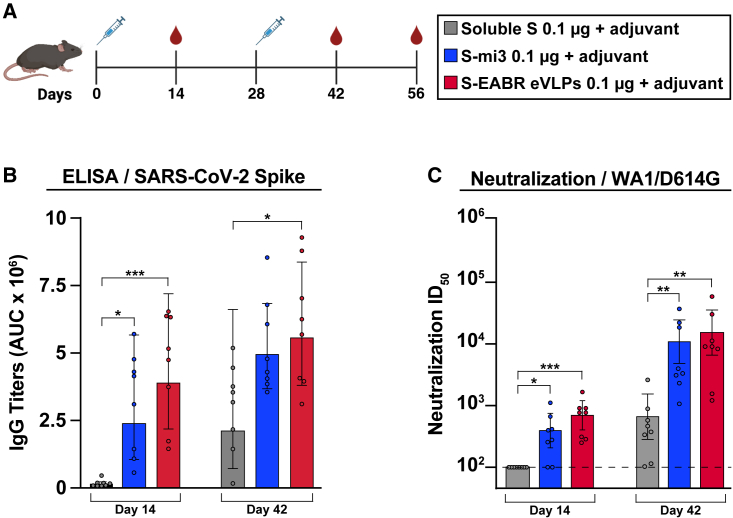
Purified S-EABR eVLPs induce potent antibody responses in mice (A) Immunization schedule. C57BL/6 mice were immunized with soluble S (purified S trimer) (gray), S-mi3 (S trimer ectodomains covalently attached to mi3, a 60-mer protein nanoparticle) (blue), or S-EABR eVLPs (red). (B and C) ELISA and neutralization data from the indicated time points for antisera from individual mice (colored circles) presented as the geometric mean (bars) and standard deviation (horizontal lines). ELISA results are shown as area under the curve (AUC); neutralization results are shown as half-maximal inhibitory dilutions (ID_50_ values). Dashed horizontal lines correspond to the background values representing the limit of detection for neutralization assays. Significant differences between cohorts linked by horizontal lines are indicated by asterisks: ^∗^p < 0.05; ^∗∗^p < 0.01; ^∗∗∗^p < 0.001. See also Figure S2.

**Figure S2:**
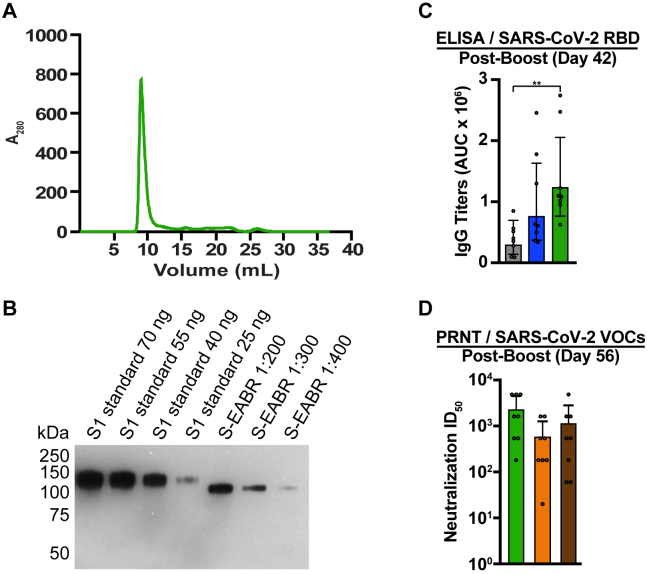
Purified S-EABR eVLPs induce potent antibody responses in mice, related to Figure 2 (A) Size exclusion chromatogram of S-EABR eVLPs purified by ultracentrifugation on a 20% sucrose cushion. (B) Quantitative western blot comparing indicated amounts of SARS-CoV-2 S1 standards (lanes 1–4) and various dilutions of purified S-EABR eVLPs (lanes 5–7) to determine S protein concentrations in eVLP samples. The S1 standard protein (Sino Biological) was biotinylated and contained a polyhistidine tag, which resulted in a difference in apparent molecular weights for the S1 standards and the S-EABR construct. Band intensities of S1 standards and S-EABR eVLP sample dilutions were measured using ImageJ to determine S concentrations. (C) ELISA data from day 42 for antisera from individual mice (colored circles) immunized with soluble S (purified S trimer) (gray), S-mi3 (S trimer ectodomains covalently attached to mi3, a 60-mer protein nanoparticle) (blue), or S-EABR eVLPs (green). Results are shown as area under the curve (AUC) and presented as the geometric mean (bars) and standard deviation (horizontal lines). Significant differences between cohorts linked by horizontal lines are indicated by asterisks: ^∗^p < 0.05; ^∗∗^p < 0.01. (D) PRNT assay results from day 56 for antisera from individual mice (colored circles) immunized with S-EABR eVLPs. Results against the SARS-CoV-2 WA1 (green), Beta (orange), and Delta (brown) variants are shown as TCID_50_ values^72^ and presented as the geometric mean (bars) and standard deviation (horizontal lines).

The potential of purified S-EABR eVLPs as a vaccine candidate against SARS-CoV-2 was evaluated in C57BL/6 mice (Figure 2A). S-EABR eVLPs were purified from transfected cell supernatants by ultracentrifugation on a 20% sucrose cushion followed by size exclusion chromatography (SEC), and S protein concentrations were determined by quantitative western blot analysis (Figures S2A and S2B). Immunizations with S-EABR eVLPs were compared with purified soluble S and to soluble S covalently attached to SpyCatcher-mi3 protein nanoparticles (S-mi3).^34^ 0.1 μg doses (calculated based on S protein content) were administered by subcutaneous injections on days 0 and 28 for all immunogens in the presence of Sigma adjuvant (Figure 2A), and we evaluated serum antibody responses by enzyme-linked immunosorbent assays (ELISAs) and *in vitro* pseudovirus neutralization assays. After the prime, S-EABR eVLPs elicited robust antibody binding and neutralization responses in all mice against SARS-CoV-2 (WA1 variant including the D614G substitution [WA1/D614G]), similar to titers elicited by S-mi3 (Figures 2B and 2C). In contrast, no neutralizing antibody responses were detected for soluble S protein immunization after the prime. Neutralizing antibody titers elicited by S-EABR eVLPs and S-mi3 increased by >10-fold after boosting and were >20-fold higher than titers measured for soluble S (Figure 2C). S-EABR eVLPs elicited potent antibody responses targeting the receptor-binding domain (RBD) of the S protein (Figure S2C), a primary target of anti-SARS-CoV-2 neutralizing antibodies.^35^ Serum responses were also evaluated against authentic SARS-CoV-2 by plaque reduction neutralization tests (PRNTs), showing robust neutralizing activity against SARS-CoV-2 WA1 (Figure S2D). Neutralization titers dropped ∼4- and ∼2-fold against the SARS-CoV-2 Beta and Delta variants, respectively, consistent with studies of licensed vaccines that encode the SARS-CoV-2 WA1 S protein.^36^ These results demonstrate that purified S-EABR eVLPs elicit potent immune responses *in vivo* and represent an alternative technology for producing nanoparticle-based vaccines that does not involve detergent-mediated cell lysis and separation of membrane protein antigens from cell lysates, as required for protein nanoparticle vaccines such as NVX-CoV2373, a COVID-19 vaccine,^9^^,^^10^ or FluBlok, an influenza vaccine.^37^

### mRNA-encoded S-EABR construct induces cell surface expression and eVLP budding

**Figure 3:**
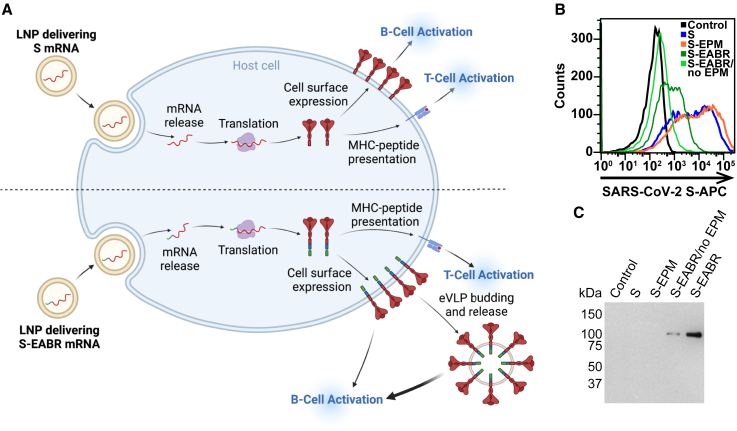
mRNA-mediated delivery of the S-EABR construct results in cell surface expression and eVLP assembly (A) Schematic comparison of mRNA-LNP delivery of S (as in COVID-19 mRNA vaccines) (top) versus delivery of an S-EABR construct (bottom). Both approaches generate S peptides displayed on class I MHC molecules for CD8^+^ T cell recognition and result in presentation of S antigens on cell surfaces. The S-EABR approach also results in budding and release of eVLPs displaying S antigens. (B) Flow cytometry analysis of SARS-CoV-2 S cell surface expression on HEK293T cells that were untransfected (black) or transfected with mRNAs encoding S (blue), S-EPM (orange), S-EABR (dark green), or S-EABR/no EPM (light green) constructs. (C) Western blot analysis of eVLPs purified by ultracentrifugation on a 20% sucrose cushion from supernatants from the transfected cells in (B). Purified eVLP samples were diluted 1:10.

A key advantage of the EABR eVLP technology over existing nanoparticle-based vaccine approaches is that S-EABR constructs can be easily delivered as mRNA vaccines since both eVLP assembly and cell surface expression only require expression of a single genetically encoded component. While conventional COVID-19 mRNA vaccines induce antibody responses through cell surface expression of S protein (Figure 3A, top), mRNA-mediated delivery of an S-EABR construct could enhance B cell activation because S-EABR proteins will not only be expressed at the cell surface—they will also induce assembly of eVLPs that bud from the cell and distribute inside the body to activate immune cells (Figure 3A, bottom).

To investigate whether genetic encoding of S-EABR eVLPs enhances the potency of a SARS-CoV-2 S-based mRNA vaccine, we started by synthesizing nucleoside-modified mRNAs encoding S, S-EABR, S-EPM, or S-EABR/no EPM. Cell surface expression and eVLP assembly were evaluated by flow cytometry and western blot analysis 48 h after *in vitro* transfection of mRNAs in HEK293T cells, demonstrating higher surface expression for S compared with the S-EABR fusion protein (Figure 3B). While addition of the EPM had little effect on S surface expression, removal of the EPM lowered surface levels for the S-EABR construct. Western blot analysis of supernatants confirmed that the S and S-EPM transfections did not generate detectable eVLPs in supernatants, whereas eVLPs were strongly detected in supernatants from S-EABR transfected cells (Figure 3C). eVLP production was decreased for S-EABR/no EPM, which, together with the flow cytometry results (Figure 3B), suggests that EPM addition enhances both S-EABR cell surface expression and eVLP assembly.

The observed reduction in S cell surface expression in the S-EABR versus S mRNA transfections could be caused by lower overall cell surface expression of the S-EABR fusion protein, incorporation of S-EABR proteins into eVLPs that bud from the cell surface, or both. To evaluate these possibilities, we calculated approximate numbers of S trimers expressed from the S-EABR construct. Assuming that 3 × 10^6^ cells were transfected (6-well plate) and up to 1 × 10^5^ S trimers were expressed on the surface of each cell (based on the approximate number of B cell receptors on a B cell^38^), transfected cell surfaces would contain ∼0.5 pmol or ∼70 ng of total S protein. Supernatant samples for western blots were concentrated to a final volume of 200 μL of which 1.2 μL was loaded onto a gel. As the detection limit for S1 is ∼20 ng, the western blot analysis suggested that purified S-EABR eVLPs from transfected cell supernatants contained at least ∼17 ng/μL S protein, corresponding to >3 μg S protein in the purified transfected cell supernatant. These calculations suggested that the observed reduction in cell surface expression for the S-EABR construct was at least partially caused by incorporation of S-EABR proteins into budding eVLPs that were released into the supernatant. Given that the estimated S protein content on released eVLPs exceeded the approximate amount of S protein presented on cell surfaces, it is possible that the S-EABR construct induces higher overall expression of S antigens compared with S for which expression is restricted to cell surfaces. Taken together, the mRNA transfection results demonstrate that the mRNA-encoded S-EABR construct enables dual presentation of S antigens on cell surfaces and released eVLPs.

### S-EABR mRNA-LNP elicit superior antibody titers compared with conventional vaccines

**Figure 4:**
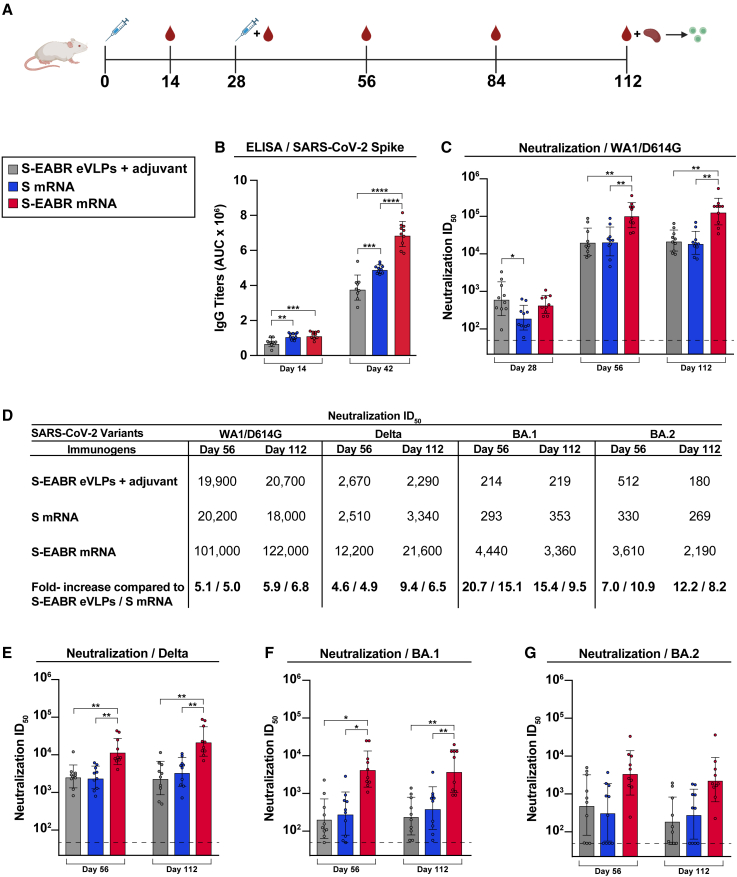
mRNA-LNP encoding S-EABR eVLPs induce potent antibody responses in mice (A) Immunization schedule. BALB/c mice were immunized with purified S-EABR eVLPs (1 μg S protein) plus adjuvant (gray), 2 μg of mRNA-LNP encoding S (blue), or 2 μg of mRNA-LNP encoding S-EABR (red). On day 112, spleens were harvested from immunized mice for ELISpot analysis. (B) ELISA data from the indicated time points for antisera from individual mice (colored circles) presented as the geometric mean (bars) and standard deviation (horizontal lines). ELISAs evaluated binding of SARS-CoV-2 S trimers; results are shown as area under the curve (AUC). (C) Neutralization data from the indicated time points for antisera from individual mice (colored circles) presented as the geometric mean (bars) and standard deviation (horizontal lines). Neutralization results against SARS-CoV-2 WA1/D614G pseudovirus are shown as geometric mean half-maximal inhibitory dilutions (ID_50_ values). Dashed horizontal lines correspond to the background values representing the limit of detection for neutralization assays. (D) Neutralization data from indicated time points for antisera presented as ID_50_ values against SARS-CoV-2 WA1/D614G, Delta, Omicron BA.1, and Omicron BA.2 pseudoviruses. Bottom horizontal row shows the fold increases for geometric mean ID_50_ values for mice that received S-EABR mRNA-LNP compared with mice that received purified S-EABR eVLPs or S mRNA-LNP. (E–G) Neutralization data from the indicated time points for antisera from individual mice (colored circles) presented as the geometric mean (bars) and standard deviation (horizontal lines). Neutralization results against SARS-CoV-2 Delta (E), Omicron BA.1 (F), and Omicron BA.2 (G) pseudoviruses are shown as ID_50_ values. Dashed horizontal lines correspond to the background values representing the limit of detection for neutralization assays. Significant differences between cohorts linked by horizontal lines are indicated by asterisks: ^∗^p < 0.05; ^∗∗^p < 0.01; ^∗∗∗^p < 0.001; ^∗∗∗∗^p < 0.0001.

The effect of eVLP production on mRNA vaccine potency was evaluated in BALB/c mice by comparing mRNAs encoding S or S-EABR constructs that were encapsulated in LNP (Figure 4A). As described for preclinical studies of a COVID-19 mRNA vaccine in mice,^1^ mRNA-LNP were administered intramuscularly (IM) at a dose of 2 μg mRNA on days 0 and 28. mRNA-LNP immunizations were also compared with purified S-EABR eVLPs that were injected IM in the presence of Addavax adjuvant. After the prime, S and S-EABR mRNA-LNP elicited significantly higher antibody binding responses against the SARS-CoV-2 S protein than purified S-EABR eVLPs (Figure 4B). However, the highest neutralizing antibody titers were elicited by purified S-EABR eVLPs, which were significantly higher than titers elicited by the S mRNA-LNP (Figure 4C).

After a boost immunization, S-EABR mRNA-LNP elicited significantly higher binding and neutralizing antibody titers than purified S-EABR eVLPs and S mRNA-LNP (Figures 4B–4D). Geometric mean neutralization titers measured for S-EABR mRNA-LNP were 5.1- and 5-fold higher than titers elicited by purified S-EABR eVLPs and S mRNA-LNP, respectively (Figures 4C and 4D). 3 months post-boost (day 112), mean neutralization titers were 5.9- and 6.8-fold higher for S-EABR mRNA-LNP compared with purified S-EABR eVLPs and S mRNA-LNP, respectively, demonstrating that the increased serum neutralization activity was maintained (Figures 4C and 4D).

We also evaluated serum neutralizing activity against SARS-CoV-2 VOCs. S-EABR mRNA-LNP elicited 4.9- and 6.5-fold higher mean neutralizing responses against the Delta variant compared with S mRNA-LNP, as well as 4.6- and 9.4-fold higher titers compared with purified S-EABR eVLPs on days 56 and 112, respectively (Figures 4D and 4E). Against Omicron BA.1, neutralizing antibody responses dropped markedly for all groups, except for mice that received S-EABR mRNA-LNP, which elicited 15.1- and 9.5-fold higher neutralizing titers than S mRNA-LNP and 20.7- and 15.4-fold higher titers than purified S-EABR eVLPs on days 56 and 112, respectively (Figures 4D and 4F). Against Omicron BA.2, mean neutralization titers measured for mice that received S-EABR mRNA-LNP were also 10.9- and 8.2-fold higher compared with S mRNA-LNP and 7- and 12.2-fold higher compared with purified S-EABR eVLPs on days 56 and 112, respectively, but these differences narrowly failed to reach statistical significance (Figures 4D and 4G). Together, these results demonstrate that mRNA-mediated delivery of S-EABR eVLPs enhances the potency and breadth of humoral immune responses in mice compared with conventional mRNA and protein nanoparticle-based vaccine approaches. The observed improvements in neutralizing activity against Omicron-based VOCs were substantially larger than the 1.5-fold increases reported for recently approved bivalent mRNA booster shots,^39^ suggesting that S-EABR mRNA-LNP-based booster immunizations could induce more effective and lasting immunity against Omicron-based and emerging VOCs than current COVID-19 vaccines.

### S-EABR mRNA-LNP induce potent T cell responses

**Figure 5:**
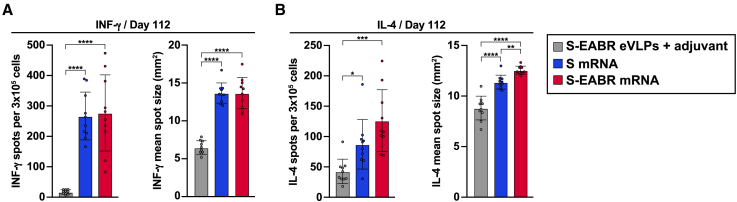
mRNA-LNP encoding S-EABR eVLPs induce potent T cell responses in mice (A and B) ELISpot assay data for SARS-CoV-2 S-specific INF-γ (A) and IL-4 (B) responses of splenocytes from BALB/c mice that were immunized with purified S-EABR eVLPs (1 μg S protein) plus adjuvant (gray), 2 μg of mRNA-LNP encoding S (blue), or 2 μg of mRNA-LNP encoding S-EABR (red). Results are shown as spots per 3 × 10^5^ cells (left) and mean spot sizes (right) for individual mice (colored circles) presented as the mean (bars) and standard deviation (horizontal lines). Significant differences between cohorts linked by horizontal lines are indicated by asterisks: ^∗^p < 0.05; ^∗∗^p < 0.01; ^∗∗∗^p < 0.001; ^∗∗∗∗^p < 0.0001.

On day 112 (3 months post-boost), splenocytes were isolated from immunized mice to analyze T cell responses by enzyme-linked immunosorbent spot (ELISpot) assays.^40^ Splenocytes were stimulated with a pool of SARS-CoV-2 S-specific peptides, and INF-γ and IL-4 secretion were measured to evaluate T cell activation. mRNA-LNP encoding S and S-EABR constructs induced potent INF-γ responses, consistent with the presence of S-specific cytotoxic CD8^+^ T cells and T helper 1 (T_H_1) cellular immune responses (Figure 5A). In contrast, INF-γ responses were almost undetectable for mice immunized with purified S-EABR eVLPs (Figure 5A). These results were expected as mRNA-LNP immunizations result in intracellular expression of S or S-EABR immunogens and MHC class I presentation of antigenic peptides that activate CD8^+^ T cells, which does not commonly occur for protein nanoparticle-based vaccines.^8^

S-EABR mRNA-LNP induced significantly stronger IL-4 responses compared with S mRNA-LNP and purified S-EABR eVLPs (Figure 5B), consistent with potent T_H_2 cellular immune responses. While T_H_1- and T_H_2-biased responses were observed for S mRNA-LNP and purified S-EABR eVLPs, respectively, S-EABR mRNA-LNP induced a balanced T_H_1/T_H_2 response, thereby potently stimulating cellular and humoral immune responses. Thus, S-EABR mRNA-LNP retain the ability of conventional S mRNA-LNP to activate potent cytotoxic CD8^+^ T cell responses, while also potently activating T_H_2 CD4^+^ T cell responses to enhance humoral immune responses leading to increased antibody potency and breadth.

## Discussion

Here, we present a new technology to generate eVLPs for vaccine and other applications. The approach harnesses the ESCRT pathway that is involved in cell division and viral budding^18^^,^^19^ to drive assembly and release of eVLPs that present membrane proteins containing a cytoplasmic ESCRT-recruiting motif, the EABR sequence from the human centrosomal protein CEP55.^41^ Our results demonstrate that the EABR-based platform produces eVLPs that incorporate higher levels of membrane antigens compared with approaches that require co-expression of the antigen with viral capsid proteins such as Gag or with the SARS-CoV-2 M, N, and E proteins. Purified S-EABR eVLPs elicited potent antibody responses against SARS-CoV-2 in mice that were similar in magnitude to those elicited by a 60-mer protein nanoparticle displaying S trimers. Compared with existing protein nanoparticle-based vaccine approaches, the EABR technology exhibits attractive manufacturing properties as (1) eVLP production requires expression of only a single component, (2) transmembrane proteins are retained in their native membrane-associated conformation to ensure optimal protein expression and stability, and (3) fully assembled eVLPs can be purified directly from culture supernatants without requiring detergent-mediated cell lysis and separation of membrane protein antigens from cell lysates. The lipid bilayer surrounding eVLPs also prevents off-target antibody responses against a nanoparticle scaffold that have been reported for protein nanoparticle-based immunogens.^42^ Due to its modularity, flexibility, and versatility, the EABR technology could potentially be used to generate eVLPs presenting a wide range of surface proteins for vaccine and therapeutic applications.

To optimize the EABR technology, we evaluated several ESCRT-recruiting motifs for their ability to drive eVLP assembly, including viral late domains from EIAV, HIV-1, and EBOV. The EABR from CEP55 generated eVLPs 10-fold more efficiently than the EIAV late domain p9. The EABR binds to ESCRT proteins ALIX and TSG101,^17^ while p9 binds only to ALIX,^23^ suggesting that efficient eVLP assembly requires recruitment of both proteins. HIV-1 p6 contains motifs that interact with both TSG101 and ALIX,^23^^,^^25^ but S-p6 constructs did not induce detectable eVLP budding in our experiments, perhaps because reported affinities are relatively low^23^^,^^43^ compared with TSG101 and ALIX affinities reported for the EABR.^17^ eVLP production might be optimized by designing ESCRT-binding motifs with increased affinities for ESCRT proteins. We were able to enhance eVLP production by including an EPM derived from the FcgRII-B1 cytoplasmic tail^44^ to reduce endocytosis of EABR-fusion proteins, which increased S-EABR cell surface expression and eVLP production.

An advantage of the EABR technology is that constructs can be easily delivered as mRNA vaccines since eVLP assembly requires expression of only a single component. This strategy results in presentation of viral surface antigens on the cell surface and on released eVLPs that could distribute throughout the body, thereby combining immune responses elicited by both conventional mRNA and protein nanoparticle-based vaccines. S-EABR mRNA-LNP elicited significantly higher binding and neutralizing antibody responses compared with conventional S-based mRNA-LNP analogous to current COVID-19 mRNA vaccines and to purified S-EABR eVLPs, suggesting that dual presentation of viral surface antigens on cell surfaces and eVLPs potentiates B cell activation. Presentation of viral surface antigens on cell surfaces alone potentially restricts expression for conventional mRNA vaccines due to a finite, and presumably limited, environment for insertion of both delivered and endogenous membrane proteins. Thus, combining cell surface expression and eVLP release for the S-EABR mRNA-LNP may increase overall presentation of viral surface antigens to the immune system. It is also possible that mRNA-mediated S-EABR eVLP production expands the biodistribution of viral surface antigens to more effectively engage B cells in lymph nodes distant from the injection site. The enhanced humoral immune responses elicited by S-EABR mRNA-LNP were consistent with potent T_H_2 cellular responses observed in S-EABR mRNA-LNP-immunized mice, which were more pronounced than in mice immunized with S mRNA-LNP or purified S-EABR eVLPs. Importantly, cytotoxic CD8^+^ T cell responses were maintained in S-EABR mRNA-LNP compared with S mRNA-LNP-immunized animals. Thus, S-EABR mRNA-LNP potently stimulate both cellular and humoral immune responses.

The higher peak antibody levels elicited by the S-EABR mRNA-LNP would likely impact the durability of protective antibody responses. Notably, differences in serum antibody titers across different immunizations were maintained until 3 months post-boost, suggesting that antibody levels might contract at similar rates for the tested vaccine types. Hence, the elevated peak antibody titers elicited by the S-EABR mRNA-LNP could result in markedly prolonged periods of immune protection compared with conventional vaccine approaches, which could minimize the need for frequent booster immunizations. Long-term studies that monitor antibody levels for several months are needed to elucidate the relationship between peak antibody titers and durability of responses.

Two immunizations with S-EABR mRNA-LNP also elicited potent neutralizing antibody responses against SARS-CoV-2 Delta and Omicron-based VOCs, suggesting that higher antibody responses could lead to enhanced protection against viral escape variants. The conventional S-based mRNA-LNP immunization only elicited weak responses against Omicron-based VOCs, consistent with outcomes reported in humans in which weak Omicron-specific responses to WA1-based vaccines were enhanced after a 3^rd^ immunization.^13^^,^^45^ S-EABR mRNA-LNP elicited >10-fold higher neutralizing antibody titers against Omicron BA.1 and BA.2 VOCs compared with S mRNA-LNP after only two immunizations, suggesting that the simple addition of a short EABR-encoding sequence to the spike gene in current mRNA vaccines could have limited the global spread of Omicron-based VOCs. Our results also suggest that S-EABR mRNA-LNP-based booster immunizations would induce superior immunity against Omicron-based and emerging VOCs compared with current boosting strategies, as bivalent booster shots that contain ancestral and Omicron-based variants improve neutralizing antibody titers by only 1.5-fold compared with conventional booster shots.^39^ Future studies need to investigate whether the observed increase in neutralization activity against Omicron-based VOCs results from higher overall antibody levels and/or increased antibody targeting of sub-immunodominant conserved epitopes on S trimer.

Enhanced antibody responses compared with S mRNA-LNP have also been reported for co-delivery of mRNAs encoding SARS-CoV-2 S, M, and E proteins, which should result in dual presentation of S on cell surfaces and released eVLPs.^46^ However, higher mRNA doses (10 μg) were needed to deliver all three mRNAs, and only modest improvements (∼2.5-fold) in neutralizing antibody titers were achieved. Our results showed that S-EABR eVLPs assemble more efficiently *in vitro* than eVLPs driven by co-expression of S, M, N, and E proteins, potentially explaining why S-EABR mRNA-LNP induced larger increases in antibody titers at lower doses. Co-delivery of multiple mRNAs also poses an obstacle for vaccine manufacturing, whereas COVID-19 and other mRNA vaccines could be easily modified to generate eVLPs by adding a short sequence containing EABR and EPM motifs to the cytoplasmic domains of the encoded immunogens. mRNA delivery of a trimerized RBD-ferritin fusion construct, which should result in secretion of non-enveloped ferritin nanoparticles displaying trimeric RBDs without cell surface expression of RBDs, has also been reported.^47^ This approach was not compared with a conventional S mRNA-LNP-based immunogen, highlighting the need for comparison studies of different vaccine approaches to elucidate the individual effects of antigen presentation on cell surfaces and virus-like nanoparticles on the magnitude and quality of immune responses.

In summary, we present a new technology to efficiently generate eVLPs for vaccine and other therapeutic applications. We demonstrate that an mRNA vaccine encoding SARS-CoV-2 S-EABR eVLPs elicits antibody responses with enhanced potency and breadth compared with conventional vaccine strategies in mice, which warrants further investigation in other preclinical animal models and humans as a vaccine strategy.

### Limitations of the study

Since our study involves immunization studies performed in mice, future studies will need to evaluate whether S-EABR mRNA immunizations also elicit more potent and broad antibody responses in non-human primates and humans compared with conventional mRNA vaccine strategies. Because binding and neutralizing antibody responses correlate with protection in humans and animals vaccinated with COVID-19 mRNA vaccines,^48^^,^^49^^,^^50^ the strong antibody responses elicited by S-EABR mRNA immunizations are predictive of protection. However, viral challenge studies in animals could provide further evidence that S-EABR mRNA immunizations induce more effective protection against Omicron-based variants. In addition, although *in vitro* experiments showed that S-EABR protein is presented on cell surfaces and on released eVLPs, we have no direct evidence that mRNA-encoded delivery of the S-EABR construct resulted in eVLP production *in vivo*. Thus, future studies are needed to confirm eVLP production and distribution *in vivo* and investigate how released eVLPs affect immune cell activation. The effects of S-EABR mRNA immunizations on T cells, Fc-mediated effector functions, and other aspects of the immune response are also needed to fully assess the potential of the EABR vaccine approach. Finally, the effectiveness of the EABR vaccine platform against other viral pathogens needs to be evaluated.

## STAR★Methods

### Key resources table

**Table:** 

REAGENT or RESOURCE	SOURCE	IDENTIFIER
**Antibodies**		

SARS/SARS-CoV-2 Coronavirus Spike Protein (subunit 1) polyclonal antibody	Thermo Fisher Scientific	Cat# PA5-81795 RRID:AB_2788969
10-1074 monoclonal antibody	Mouquet et al.^51^	https://www.pnas.org/doi/full/10.1073/pnas.1217207109; RRID:AB_2491062
CCR5 monoclonal antibody	Abcam	Cat# ab111300, RRID:AB_10863746
Peroxidase IgG Fraction Monoclonal Mouse Anti-Rabbit IgG, light chain specific	Jackson ImmunoResearch	Cat# 211-032-171, RRID:AB_2339149
Goat Anti-Human IgG Fc, Multi-Species SP ads-HRP	SouthernBiotech	Cat# 2014-05, RRID:AB_2795580
Mouse Anti-Rat IgG2a-HRP (2A8F4)	SouthernBiotech	Cat# 3065-05, RRID:AB_2795873
HRP-conjugated goat anti-mouse IgG	Jackson ImmunoResearch	Cat# 715-035-150 RRID:AB_2340770
C119 monoclonal antibody	Robbiani et al.^52^	https://doi.org/10.1038/s41586-020-2456-9
Goat anti-Human IgG (H+L) Cross-Adsorbed Secondary Antibody, Alexa Fluor™ 647	Invitrogen	Cat# A-21445 RRID:AB_2535862

**Bacterial and virus strains**		

*E. coli* DH5 Alpha	Zymo Research	Cat# T3009
*E. coli* BL21-CodonPlus (DE3)-RIPL	Agilent Technology	Cat# 230280
SARS-CoV-2 pseudotyped reporter virus	BEI	Cat# NR-53817
SARS-CoV-2 Delta pseudotyped reporter virus	Cohen et al.	https://www.science.org/10.1126/science.abq0839
SARS-CoV-2 Omicron BA.1 pseudotyped reporter virus	Cohen et al.	https://www.science.org/10.1126/science.abq0839
SARS-CoV-2 Omicron BA.2 pseudotyped reporter virus	Cohen et al.	https://www.science.org/10.1126/science.abq0839
wild-type SARS-CoV-2 USA-WA1/2020	BEI Resources	Cat# NR-52281
Beta variant, isolate hCoV-19/South Africa/KRISP-K005325/2020	BEI Resources	Cat# NR-54009
Delta variant, isolate hCoV-19/USA/MD-HP05647/2021	BEI Resources	Cat# NR-55674

**Chemicals, peptides, and recombinant proteins**		

LB Broth (Miller)	Sigma-Aldrich	Cat# L3522
2xYT media	Sigma-Aldrich	Cat# Y2377-250G
IPTG	RPI	Cat# I56000-100.0
Dulbecco’s Modified Eagle Medium (DMEM)	Gibco	Cat# 11995073
Fetal bovine serum (FBS)	Sigma-Aldrich	Cat# F4135
Penicillin-Streptomycin	Gibco	Cat# 15070063
FuGENE HD Transfection Reagent	Promega	Cat# E2311
Expi293 Expression Medium	Gibco	Cat# A1435102
Expi293 Expression System Kit	Gibco	Cat# A14635
Phosphate-Buffered Saline (10X) pH 7.4, RNase-free	Invitrogen	Cat# AM9625
Lipofectamine™ MessengerMax™ transfection reagent	Thermo Fisher Scientific	Cat# LMRNA008
Sucrose	SAFC	Cat# ARK2195B
PMSF	Sigma-Aldrich	Cat# 52332
SARS-CoV-2 (2019-nCoV) Spike S1-His Recombinant Protein, Biotinylated	SinoBiological	Cat# 40591-V08H-B
BriteLite Plus Substrate	Perkin Elmer	Cat# 6066769
TWEEN 20	Sigma-Aldrich	Cat# P1379
BSA	Sigma-Aldrich	Cat# 03116956001
SuperSignal ELISA Femto Maximum Sensitivity Substrate	Thermo Fisher Scientific	Cat# 37074
Methanol	Sigma-Aldrich	Cat# 34860
Methylcellulose	Sigma-Aldrich	Cat# M0512
Crystal violet	Sigma-Aldrich	Cat# HT901
Sigma Adjuvant System®	Sigma-Aldrich	Cat# S6322-1VL
AddaVax™ Adjuvant	InvivoGen	Cat# vac-adx-10
CTL-Test™ media	ImmunoSpot	Cat# CTLT-010
GlutaMAX™ supplement	Gibco	Cat# 35050061

**Critical commercial assays**		

Amersham ECL Prime Western Blotting Detection Reagent	Cytiva	Cat# RPN2232
Mouse IFN-γ/IL-4 Double-Color ELISPOT	ImmunoSpot	Cat# mIFNgIL4-1M
Luciferase Cell Culture Lysis 5X Reagent	Promega	Cat#E1531
Nano-Glo Luciferase Assay System	Promega	Cat# N1110
Quant-iT Ribogreen Assay	Invitrogen	Cat# R11490
HEK293T cells	^53^	RRID:CVCL_0063
Expi293F cells	Gibco	RRID:CVCL_D615
HEK293T-ACE2 cells	BEI Resources	Cat# NR-52511
Vero/TMPRSS2 cells	Adrian Creanga, VRC, NIAID, Bethesda, MD	RRID:CVCL_YQ48

**Experimental models: Organisms/strains**		

C57BL/6 mice (7-8 week old, female)	Charles River Laboratories	N/A
BALB/c mice (7-8 week old, female)	Charles River Laboratories	N/A

**Recombinant DNA**		

p3BNC-SARS-CoV-2 S-2P-ΔCT	This paper	N/A
p3BNC-SARS-CoV-2 S-2P-ΔCT-EABR	This paper	N/A
p3BNC-SARS-CoV-2 S-2P-ΔCT-EABR/no EPM	This paper	N/A
p3BNC-SARS-CoV-2 S-2P-ΔCT-EABR_min1_	This paper	N/A
p3BNC-SARS-CoV-2 S-2P-ΔCT-EABR_min2_	This paper	N/A
p3BNC-HIV-1 Env_YU2_-EABR	This paper	N/A
p3BNC-human CCR5-EABR	This paper	N/A
p3BNC-SARS-CoV-2 S-2P-ΔCT-EIAV p9	This paper	N/A
p3BNC-SARS-CoV-2 S-2P-ΔCT-EBOV VP40_1-44_	This paper	N/A
p3BNC-SARS-CoV-2 S-2P-ΔCT-HIV-1 p6	This paper	N/A
p3BNC-SARS-CoV-2 S-2P-ΔCT-2xEABR	This paper	N/A
p3BNC-SARS-CoV-2 S-2P-ΔCT-EABR_mut_	This paper	N/A
p3BNC-SARS-CoV-2 S-2P (full CT)	This paper	N/A
p3BNC-SARS-CoV-2 M	This paper	N/A
p3BNC-SARS-CoV-2 N	This paper	N/A
p3BNC-SARS-CoV-2 E	This paper	N/A
p3BNC-SARS-CoV-2 S-2P-Ferritin	This paper	N/A
pHDM-Hgpm2	PlasmID Repository, Harvard Medical School	N/A
SARS-CoV-2 S-6P-SpyTag003	This paper	N/A
SpyCatcher003-mi3 expression plasmid	Addgene	RRID:Addgene_159995
pcDNA-BirA expression plasmid	Gift, Michael Anaya (Caltech)	N/A

**Software and algorithms**		

Image J	Rueden et al., 2017^54^	https://imagej.net/ RRID:SCR_003070
SerialEM 3.7	Mastrondarde^55^	https://pubmed.ncbi.nlm.nih.gov/16182563/
IMOD	Mastronarde and Held^56^	https://bio3d.colorado.edu/imod/ RRID:SCR_003297
UCSF ChimeraX	Goddard et al.^57^ and Pettersen et al.^58^	https://www.cgl.ucsf.edu/chimerax/ RRID:SCR_015872
cellPACK	Johnson et al.^59^^,^^60^	http://www.cellpack.org
AntibodyDatabase	West et al.^61^	https://www.pnas.org/doi/full/10.1073/pnas.1309215110
Prism 9.3.1	GraphPad	https://www.graphpad.com/scientific-software/prism/
FlowJo 10.5.3	FlowJo	https://www.flowjo.com/solutions/flowjo
Illustrator 2022	Adobe	https://www.adobe.com/products/illustrator/

**Other**		

Amicon® Ultra-15 100 kDa MWCO Centrifugal Filter Unit	EMD Millipore	Cat# UFC910096
Amicon® Ultra-15 30 kDa MWCO Centrifugal Filter Unit	EMD Millipore	Cat# UFC903096
Amicon® Ultra-4 100 kDa MWCO Centrifugal Filter Unit	EMD Millipore	Cat# UFC810096
Amicon® Ultra-4 30 kDa MWCO Centrifugal Filter Unit	EMD Millipore	Cat# UFC803096
0.45-μm syringe filter	Corning	Cat# 431220
0.20-μm Acrodisc Sterile Syringe Filters	Pall Laboratory	Cat# 4602
HisTrap™ HP column	Cytiva	Cat# 17-5248-02
Superose 6 Increase 10/300 column	Cytiva	Cat# 29-0915-96
HiLoad 16/600 Superdex 200 column	Cytiva	Cat# 28-9893-35
Nitrocellulose, 0.45-um pore size	Thermo Fisher Scientific	Cat# LC2001
10 nm fiducial gold beads	BBI Solutions	Cat# 15703-20
Quantifoil R 2/2 300 Mesh, Gold grids	Quantifoil Micro Tools	Cat# Q350AR2
Nunc® MaxiSorp™ 384 well plates	Thermo Fisher Scientific	Cat# 464718
gentleMACS™ Octo Dissociator with Heaters	Miltenyi Biotec	Cat# 130-096-427
PepMix™ SARS-CoV-2 (Spike Glycoprotein) (Research Plus Grade)	JPT Peptide Technologies	Cat# PM-WCPV-S-1

### Resource availability

#### Lead contact

Further information and requests for resources and reagents should be directed to and will be fulfilled by the lead contacts, Magnus A.G. Hoffmann (mhoffman@caltech.edu) and Pamela J. Bjorkman (bjorkman@caltech.edu).

#### Materials availability

All expression plasmids generated in this study are available upon request through a Materials Transfer Agreement.

### Experimental model and subject details

#### Bacteria

*E. coli* DH5 Alpha cells (Zymo Research) used for expression plasmid productions were cultured in LB broth (Sigma-Aldrich) with shaking at 250 rpm at 37°C. *E. coli* BL21-CodonPlus (DE3)-RIPL cells (Agilent Technology) used for producing SpyCatcher003-mi3 were cultured in 2xYT media with shaking at 220 rpm at 37°C, IPTG was added at OD of 0.5 and induction lasted for 5 hours at 30°C.

#### Cell lines

HEK293T cells were cultured in Dulbecco’s modified Eagle’s medium (DMEM, Gibco) supplemented with 10% heat-inactivated fetal bovine serum (FBS, Sigma-Aldrich) and 1 U/ml penicillin-streptomycin (Gibco) at 37°C and 5% CO_2_ for pseudovirus production. Expi293F cells (Gibco) for protein expression were maintained at 37°C and 8% CO_2_ in Expi293 expression medium (Gibco). Transfections were carried out with an Expi293 Expression System Kit (Gibco) and maintained under shaking at 130 rpm. All cell lines were derived from female donors and were not specially authenticated.

#### Viruses

Pseudovirus stocks were generated by transfecting HEK293T cells with pNL4-3∆Env-nanoluc and SARS-CoV-2 S constructs^52^ using FuGENE HD (Promega); co-transfection of pNL4-3∆Env-nanoluc with a SARS-CoV-2 S construct will lead to the production of HIV-1-based pseudovirions carrying the coronavirus S protein at the surface. Eight hours after the transfection, cells were washed twice with phosphate buffered saline (PBS) and fresh media was added. Pseudoviruses in the supernatants were harvested 48 hours post-transfection, filtered, and stored at -80°C until use. Infectivity of pseudoviruses was determined by titration on HEK293T-ACE2 cells.

### Method details

#### Design of EABR constructs

The EABR domain (residues 160-217) of the human CEP55 protein was fused to the C-terminus of the SARS-CoV-2 S protein (WA1/D614G) separated by a 4-residue (Gly)_3_Ser (GS) linker to generate p3BNC-SARS-CoV-2 S-2P-∆CT-EABR/no EPM (S-EABR/noEPM) in the p3BNC expression plasmid. This construct contained the native furin cleavage site, 2P stabilizing mutations,^21^ and the C-terminal 21 residues were truncated to remove an ER-retention signal.^22^ The p3BNC-SARS-CoV-2 S-2P-∆CT-EABR (S-EABR) construct was generated by inserting residues 243-290 of mouse FcgRII-B1 upstream of the 4-residue GS linker and the EABR domain. The p3BNC-SARS-CoV-2 S-2P-∆CT-EABR_min1_ (S-EABR_min1_) and p3BNC-SARS-CoV-2 S-2P-∆CT-EABR_min2_ (S-EABR_min2_) constructs encoded residues 170-217 and 170-208 of CEP55, respectively. EABR constructs were also generated for HIV-1 Env_YU2_ (p3BNC-HIV-1 Env_YU2_-EABR) and human CCR5 (p3BNC-CCR5-EABR). p3BNC-SARS-CoV-2 S-2P-∆CT-HIV-1 p6 (S-p6), p3BNC-SARS-CoV-2 S-2P-∆CT-EBOV VP40_1-44_ (S-EBOV VP40_1-44_), and p3BNC-SARS-CoV-2 S-2P-∆CT-EIAV p9 (S-p9)were generated by replacing the EABR domain with sequences encoding HIV-1 p6 (isolate HXB2), EBOV VP40 (residues 1-44; Zaire EBOV), and EIAV p9 (strain Wyoming), respectively. The p3BNC-SARS-CoV-2 S-2P-Ferritin (S-Ferritin) construct was designed as described^31^ by fusing genes encoding the ectodomain of SARS-CoV-2 S WA1/D614G containing a furin cleavage site and 2P mutations, and *Helicobacter pylori* ferritin, separated by a 3-residue Ser-Gly-Gly linker.

#### Production of EABR eVLPs

EABR eVLPs were generated by transfecting Expi293F cells (Gibco) cultured in Expi293F expression media (Gibco) on an orbital shaker at 37°C and 8% CO_2_. Gag-based eVLPs were produced by co-transfecting Expi293F cells with a plasmid expressing Rev-independent HIV-1 Gag-Pol (pHDM-Hgpm2 plasmid; PlasmID Repository, Harvard Medical School) and SARS-CoV-2 S, HIV-1 Env_YU2_, or CCR5, respectively, at a ratio of 1:1. SARS-CoV-2 M/N/E-based eVLPs were produced by co-transfecting Expi293F cells with plasmids expressing the SARS-CoV-2 M, N, E, and S proteins at a ratio of 1:1:1:1. To enable interactions between M, N, E, and S, we transfected full-length S with an untruncated cytoplasmic domain. 72 hours post-transfection, cells were centrifuged at 400 x g for 10 min, supernatants were passed through a 0.45 μm syringe filter and concentrated using Amicon Ultra-15 centrifugal filters with 100 kDa molecular weight cut-off (Millipore). eVLPs were purified by ultracentrifugation at 50,000 rpm (135,000 x g) for 2 hours at 4°C using a TLA100.3 rotor and an Optima™ TLX ultracentrifuge (Beckman Coulter) on a 20% w/v sucrose cushion. Supernatants were removed and pellets were re-suspended in 200 μL sterile PBS at 4°C overnight. To remove residual cell debris, samples were centrifuged at 10,000 x g for 10 min and supernatants were collected. For in vivo studies and cryo-ET, eVLPs were further purified by SEC using a Superose 6 Increase 10/300 column (Cytiva) equilibrated with PBS. Peak fractions corresponding to S-EABR eVLPs were combined and concentrated to 250-500 μL in Amicon Ultra-4 centrifugal filters with 100 kDa molecular weight cut-off. Samples were aliquoted and stored at -20°C.

#### Protein expression

Soluble SARS-CoV-2 S-6P trimers (WA1/D614G)^62^ and RBDs were expressed as described.^63^^,^^64^ Briefly, Avi/His-tagged proteins were purified from transiently-transfected Expi293F cells (Gibco) by nickel affinity chromatography (HisTrap HP, Cytiva) and SEC (Superose 6 Increase 10/300, Cytiva).^63^^,^^64^^,^^65^ Peak fractions corresponding to S-6P or RBD proteins were pooled, concentrated, and stored at 4°C. Biotinylated proteins for ELISAs were generated by co-transfection of Avi/His-tagged S-6P and RBD constructs with a plasmid encoding an endoplasmic reticulum-directed BirA enzyme (kind gift from Michael Anaya, Caltech). S-6P constructs with a C-terminal SpyTag003 tag^34^ were expressed for covalent coupling to a 60-mer protein nanoparticle (SpyCatcher003-mi3) using the SpyCatcher-SpyTag system.^66^^,^^67^

#### Preparation of SpyCatcher003-mi3 nanoparticles

SpyCatcher003-mi3^68^ displaying SpyTagged SARS-CoV-2 S-6P trimers were prepared as described.^63^^,^^68^ Briefly, SpyCatcher003-mi3 subunits with N-terminal 6xHis tags were expressed in BL21-CodonPlus (DE3)-RIPL *E. coli* (Agilent). Bacterial cell pellets were lysed using a cell disruptor in the presence of 2.0 mM PMSF (Sigma). Lysates were centrifuged at 21,000 x g for 30 min, and supernatants were collected and filtered through a 0.2 μm filter. SpyCatcher003-mi3 was purified by Ni-NTA chromatography using a pre-packed HisTrap™ HP column (Cytiva), concentrated in Amicon Ultra-15 centrifugal filters with 30 kDa molecular weight cut-off (Millipore), and purified by SEC on a HiLoad 16/600 Superdex 200 column (GE Healthcare) equilibrated with TBS. S-mi3 nanoparticles were generated by incubating purified SpyCatcher003-mi3 with a 3-fold molar excess of purified SpyTagged S-6P trimer overnight at 4°C in TBS. Conjugated S-mi3 nanoparticles were separated from uncoupled S-6P trimers by SEC using a Superose 6 Increase 10/300 column (Cytiva) equilibrated with PBS (Invitrogen). Fractions corresponding to conjugated S-mi3 were identified by sodium dodecyl sulfate polyacrylamide gel electrophoresis (SDS-PAGE) and pooled.

#### Western blot analysis

The presence of SARS-CoV-2 S, HIV-1 Env_YU2_, and CCR5 on purified eVLPs was detected by Western blot analysis. Samples were diluted in SDS-PAGE loading buffer under reducing conditions, separated by SDS-PAGE, and transferred to nitrocellulose membranes (0.45 μm) (LC2001; Thermo Fisher Scientific). The following antibodies were used for detecting SARS-CoV-2 S, HIV-1 Env_YU2_, and CCR5: rabbit anti-SARS-CoV-2 S1 protein (PA5-81795; Thermo Fisher Scientific) at 1:2,500, the human anti-HIV-1 Env broadly neutralizing antibody 10-1074^51^ (expressed in-house) at 1:10,000, rat anti-CCR5 (ab111300; Abcam) at 1:2,000, HRP-conjugated mouse anti-rabbit IgG (211-032-171; Jackson ImmunoResearch) at 1:10,000, HRP-conjugated goat anti-human IgG (2014-05; Southern Biotech) at 1:8,000, and HRP-conjugated mouse anti-rat IgG (3065-05; Southern Biotech) at 1:10,000. Protein bands were visualized using ECL Prime Western Blotting Detection Reagent (RPN2232; Cytiva).

For in vivo studies, the amount of SARS-CoV-2 S on S-EABR eVLPs was determined by quantitative Western blot analysis. Various dilutions of SEC-purified S-EABR eVLP samples and known amounts of soluble SARS-CoV-2 S1 protein (40591-V08H-B-20; SinoBiological) were separated by SDS-PAGE and transferred to nitrocellulose membranes (0.45 μm) (LC2001; Thermo Fisher Scientific). SARS-CoV-2 S was detected as described above. Band intensities of the SARS-CoV-2 S1 standards and S-EABR eVLP sample dilutions were measured using ImageJ to determine S concentrations. The S1 protein concentrations determined for S-EABR eVLP samples were multiplied by a factor of 1.8 to account for the difference in molecular weight between S1 and the full-length S protein.

#### Cryo-ET of S-EABR eVLPs

SEC-purified S-EABR eVLPs were prepared on grids for cryo-ET using a Mark IV Vitrobot (Thermo Fisher Scientific) operated at 21°C and 100% humidity. 2.5 μL of sample was mixed with 0.4 μL of 10 nm fiducial gold beads (Sigma-Aldrich) and applied to 300-mesh Quantifoil R2/2 grids (GOQ300R22Cu10; Quantifoil Micro Tools), blotted for 3.5 s, and then plunge-frozen in liquid ethane cooled by liquid nitrogen. Image collections were performed on a 300 kV Titan Krios transmission electron microscope (Thermo Fisher Scientific) operating at a nominal 42,000x magnification. Tilt series were collected on a K3 direct electron detector (Gatan) with a pixel size of 2.15 Å⋅pixel^-1^ using SerialEM software.^55^ The defocus range was set to -5 to -8 μm and a total of 120 e^-^ ⋅ Å^-2^ per tilt series. Images were collected using a dose-symmetric scheme^69^ ranging from -60° to 60° with 3° intervals. Tomograms were aligned and reconstructed using IMOD.^56^

To build a model of an S-EABR eVLP, coordinates of a SARS-CoV-2 S trimer (PDB 6VXX) were fit into spike densities in the reconstructed tomograms using ChimeraX.^57^^,^^58^ Positions and orientations of the S protein were adjusted in a hemisphere of the eVLP in which the spike density was of higher quality. A 55 nm sphere was adapted from a cellPACK model (cellPACK ID: HIV-1_0.1.6_6)^59^^,^^60^ and added to the model to represent the eVLP membrane surface.

#### Neutralization assays

Lentivirus-based SARS-CoV-2 pseudoviruses were generated as described^52^^,^^70^ using S proteins from the WA1/D614G, Delta, Omicron BA.1, and Omicron BA.2 variants in which the C-terminal 21 residues of the S protein cytoplasmic tails were removed.^70^ Serum samples from immunized mice were heat-inactivated for 30 min at 56°C. Three-fold serial dilutions of heat-inactivated samples were incubated with pseudoviruses for 1 hour at 37°C, followed by addition of the serum-virus mixtures to pre-seeded HEK293T-ACE2 target cells. After 48-hour incubation at 37°C, BriteLite Plus substrate (Perkin Elmer) was added and luminescence was measured. Half-maximal inhibitory dilutions (ID_50_s) were calculated using 4-parameter non-linear regression analysis in AntibodyDatabase^61^ and ID_50_ values were rounded to three significant figures.

PRNT_50_ (50% plaque reduction neutralization test) assays with authentic SARS-CoV-2 virus were performed in a biosafety level 3 facility at BIOQUAL, Inc. (Rockville, MD) as described.^71^ Mouse sera from day 56 post-immunization were diluted 1:20 and then 3-fold serially diluted in culture media (DMEM + 10% FBS + Gentamicin). The diluted samples were incubated with 30 plaque-forming units of wild-type SARS-CoV-2 (USA-WA1/2020, BEI Resources NR-52281; Beta variant, Isolate hCoV-19/South Africa/KRISP-K005325/2020, BEI Resources NR-54009; Delta variant, isolate hCoV-19/USA/MD-HP05647/2021 BEI Resources NR-55674) for 1 hour at 37°C. Samples were then added to a confluent monolayer of Vero/TMPRSS2 cells in 24-well plates for 1 hour at 37°C in 5% CO_2_. 1 mL of culture media with 0.5% methylcellulose was added to each well and plates were incubated for 3 days at 37°C in 5% CO_2_. Plates were fixed with ice cold methanol at -20°C for 30 min. Methanol was discarded and plates were stained with 0.2% crystal violet for 30 min at room temperature. Plates were washed once with water and plaques in each well were counted. TCID_50_ values were calculated using the Reed-Muench formula.^72^

#### ELISAs

Pre-blocked streptavidin-coated Nunc® MaxiSorp™ 384-well plates (ThemoFisher Scientific) were coated with 5 μg/mL biotinylated S-6P or RBD proteins in Tris-buffered saline with 0.1% TWEEN 20 (TBS-T) and 3% bovine serum albumin (BSA) for 1 hour at room temperature. Serum samples from immunized mice were diluted 1:100, 4-fold serially diluted in TBS-T/3% BSA, and then added to plates. After a 3-hour incubation at room temperature, plates were washed with TBS-T using an automated plate washer. HRP-conjugated goat anti-mouse IgG (715-035-150; Jackson ImmunoResearch) was diluted 1:100,000 in TBS-T/3% BSA and added to plates for 1 hour at room temperature. After washing with TBS-T, plates were developed using SuperSignal™ ELISA Femto Maximal Signal Substrate (Thermo Fisher Scientific) and absorbance was measured at 425 nm. Area under the curve (AUC) calculations for binding curves were performed using GraphPad Prism 9.3.1 assuming a one-site binding model with a Hill coefficient as described.^68^

#### mRNA synthesis

Codon-optimized mRNAs encoding SARS-CoV-2 S, S-EPM, S-EABR/no EPM, and S-EABR constructs were synthesized by RNAcore (https://www.houstonmethodist.org/research-cores/rnacore/) using proprietary manufacturing protocols. mRNAs were generated by T7 RNA polymerase-mediated in vitro transcription reactions using DNA templates containing the immunogen open reading frame flanked by 5′ untranslated region (UTR) and 3′ UTR sequences and terminated by an encoded polyA tail. CleanCap 5’ cap structures (TriLink) were incorporated into the 5′ end co-transcriptionally. Uridine was completely replaced with N1-methyl-pseudouridine to reduce immunogenicity.^73^ mRNAs were purified by oligo-dT affinity purification and high-performance liquid chromatography (HPLC) to remove double-stranded RNA contaminants.^74^ Purified mRNAs were stored at –80 °C.

#### mRNA transfections

For mRNA transfections, 10^6^ HEK293T cells were seeded in 6-well plates. After 24 hours, cells were transfected with 2 μg mRNA encoding SARS-CoV-2 S, S-EPM, S-EABR/no EPM, or S-EABR constructs using Lipofectamine™ MessengerMax™ transfection reagent (Thermo Fisher Scientific). 48 hours post-transfection, supernatants were collected and purified for Western blot analysis. Cells were gently detached by pipetting and resuspended in 500 μL PBS. 100 μL were transferred into Eppendorf tubes for flow cytometry analysis of S cell surface expression. Cells were stained with the SARS-CoV-2 antibody C119^52^ at 5 μg/mL in PBS+ (PBS supplemented with 2% FBS) for 30 min at room temperature in the dark. After two washes in PBS+, samples were stained with an Alexa Fluor® 647-conjugated anti-human IgG secondary antibody (A21445; Invitrogen) at a 1:2,000 dilution in PBS+ for 30 min at room temperature in the dark. After two washes in PBS+, cells were resuspended in PBS+ and analyzed by flow cytometry (MACSQuant, Miltenyi Biotec). Results were plotted using FlowJo 10.5.3 software.

#### LNP encapsulation of mRNAs

Purified N1-methyl-pseudouridine mRNA was formulated in LNP as previously described.^75^ In brief, 1,2-distearoyl-*sn*-glycero-3-phosphocholine, cholesterol, a PEG lipid, and an ionizable cationic lipid dissolved in ethanol were rapidly mixed with an aqueous acidic solution containing mRNA using an in-line mixer. The ionizable lipid and LNP composition are described in the international patent application WO2017075531(2017). The post in-line solution was dialyzed with PBS to remove the ethanol and displace the acidic solution. Subsequently, LNP was measured for size (60-65 nm) and polydispersity (PDI < 0.075) by dynamic light scattering (Malvern Nano ZS Zetasizer). Encapsulation efficiencies were >97% as measured by the Quant-iT Ribogreen Assay (Invitrogen).

#### Immunizations

All animal procedures were performed in accordance with IACUC-approved protocols. 7-8 week-old female C57BL/6 or BALB/c mice (Charles River Laboratories) were used for immunization experiments with cohorts of 8-10 animals per group. 0.1 μg of protein-based immunogens, including soluble S trimer, S-mi3, and purified S-EABR eVLPs, were administered to C57BL/6 mice by subcutaneous (SC) injections on days 0 and 28 in the presence of Sigma adjuvant system (Sigma-Aldrich). 2 μg of S and S-EABR mRNA-LNP were administered to BALB/c mice by intramuscular (IM) injections on days 0 and 28. To compare mRNA- and protein-based immunogens, 1 μg purified S-EABR eVLPs were administered IM in the presence of 50% v/v AddaVax™ adjuvant (InvivoGen). Serum samples for ELISAs and neutralization assays were obtained on indicated days.

#### ELISpot assays

Animals were euthanized on day 112 and spleens were collected. Spleens were homogenized using a gentleMACS Octo Dissociator (Miltenyi Biotec). Cells were passed through a 70 μm tissue screen, centrifuged at 1,500 rpm for 10 min, and resuspended in CTL-Test™ media (ImmunoSpot) containing 1% GlutaMAX™ supplement (Gibco) for ELISpot analysis to evaluate T cell responses. A PepMix™ pool of 315 peptides (15-mers with 11 amino acid overlap) derived from the SARS-CoV-2 S protein (JPT Peptide Technologies) was added to mouse IFN-g/IL-4 double-color ELISpot plates (ImmunoSpot) at a concentration of 2 μg/mL. 300,000 cells were added per well, and plates were incubated at 37°C for 24 hours. Biotinylated detection, streptavidin-alkaline phosphatase (AP), and substrate solutions were added according to the manufacturer’s guidelines. Plates were gently rinsed with water three times to stop the reactions. Plates were air-dried for two hours in a running laminar flow hood. The number of spots and the mean spot sizes were quantified using a CTL ImmunoSpot S6 Universal-V Analyzer (Immunospot).

### Quantification and statistical analysis

Titer differences between immunized groups of C57BL/6 mice (8 mice per group) for ELISAs and neutralization assays were evaluated for statistical significance using the non-parametric Kruskal-Wallis test followed by Dunn’s multiple comparison post hoc test calculated using Graphpad Prism 9.3.1. Statistically significant titer differences between immunized groups of BALB/c mice (10 mice per group) for ELISAs and neutralization assays were determined using analysis of variance (ANOVA) test followed by Tukey’s multiple comparison post hoc test calculated using Graphpad Prism 9.3.1.

## Data Availability

All data are available in the main text or the supplemental information. Materials are available upon request to the corresponding authors with a signed material transfer agreement. Any additional information required to reanalyze the data reported in this paper is available from the lead contact upon request. This paper does not report original code. This work is licensed under a Creative Commons Attribution 4.0 International (CC BY 4.0) license, which permits unrestricted use, distribution, and reproduction in any medium, provided the original work is properly cited. To view a copy of this license, visit https://creativecommons.org/licenses/by/4.0/. This license does not apply to figures/photos/artwork or other content included in the article that is credited to a third party; obtain authorization from the rights holder before using such material.
